# Computerized-assisted technology of virtual reality on memory in people with Williams syndrome

**DOI:** 10.3389/fpsyg.2025.1569243

**Published:** 2025-08-27

**Authors:** Ching-Fen Hsu, Qian Jiang

**Affiliations:** ^1^School of Foreign Languages and Literature, Wuhan University, Wuhan, China; ^2^Laboratory for Language Pathology and Developmental Neurosciences, Wuhan University, Wuhan, China; ^3^School of Foreign Languages, Hunan University, Changsha, China

**Keywords:** Williams syndrome, virtual reality, long-term memory, rehabilitation potential, lexical semantics

## Abstract

**Background:**

People with Williams syndrome (WS) have strong verbal short-term memory but challenged verbal long-term memory given their advantageous lexical semantics.

**Aims:**

This study aimed to evaluate the memory of people with WS using virtual reality (VR) to determine the root of challenges associated with virtual navigation.

**Methods:**

People with WS (*n* = 20, chronological age [CA] = 12.5, mental age [MA] = 8.9) were recruited. Four typically developing control groups participated in a navigation task in a shopping setting: CA-matched (*n* = 20, mean = 12.5), MA-matched (*n* = 20, mean = 8.8), the 5th graders (*n* = 20, mean = 10.3), and college students (*n* = 20, CS, mean = 20.2). Fourteen indices were measured and error patterns were analyzed across groups.

**Results:**

Based on the shopping task, if a participant did not follow the instructions and/or target list, an error was recorded and reported using the software. People with WS shopped the longest and erred the most. The CA group shopped longer and erred more than the MA group; the 5th graders were similar to the CS group. People with WS replaced and confused more than controls. Further analyses revealed atypical processing of the semantic features of the target items in people with WS. As control groups, the practice effect emerged through pause time and duration in people with WS.

**Conclusion:**

The findings revealed that people with WS show bizarre lexical semantic knowledge, which may be an influential factor in impaired long-term memory and sentence comprehension. The practice effect seems to be an important factor in the rehabilitation potential of people with WS.

**Implications:**

VR technology could be a promising tool for assessing memory and cognitive abilities in people with WS. With computerized-assisted technological advancements in training, people with WS can improve their long-term memory and sentence comprehension abilities with a specific design and aim for the target issue.

## Highlights

- This study contributes to the potential rehabilitation of people with WS using advanced VR technology through navigation tasks.
- People with WS showed possible improvement via practice effects as their typically developing controls.
- This study identifies the possible causes of the impairment of lexical integration in sentences in people with WS.

## 1 Introduction

Williams syndrome (WS) is a rare disease characterized by genetic deficits on chromosome 7q11.23 with 15–22 genes missing (Korenberg et al., 2000). This syndrome was first identified in 1961 by Dr. William Burren, and its epidemiology is one in 25,000 live births (Semel and Rosner, 2003). Due to this genetic deficit, people with WS have intellectual disabilities, with a mean IQ of 55 (Bellugi et al., 2000). People with WS have relatively good language skills and impaired visuospatial abilities; they have normal short-term memory but impaired long-term memory (Bellugi et al., 1994). Vicari et al. (1996) conducted studies to examine the memory abilities of people with WS and revealed dissociation of verbal and spatial memory. In the digit forward span test, people with WS performed as control groups. In the spatial forward span test, people with WS were significantly worse than typically developing (TD) controls. In the verbal learning task, people with WS differed from TD in the primacy effect (1–3 digits) but were similar to TD in the recency effect (10–12 digits). This finding suggests that people with WS are deficient in long-term memory but typical in short-term memory.

Previous studies have shown advantageous phonological processing in short-term memory, leading to rich knowledge of lexical semantics in people with WS (Rossen et al., 1996; Bellugi et al., 2000). From language acquisition, vocabulary density closely relates to syntactic development. However, people with WS are impaired in sentence comprehension (Hsu and Tzeng, 2011; Hsu, 2013a, 2013b, 2013c, 2014; Neville et al., 1994). This paradox might be rooted in a superficial understanding of lexical items and problematic linkages among people with WS (Mervis, 1999; Mervis et al., 1999; Mervis and Robinson, 2003; Thomas et al., 2010; van Herwegen et al., 2013).

Virtual reality (VR) is a useful assessment tool for people with cognitive decline across different languages, such as a supermarket navigation task (English: Waterlander et al., 2011; Turkish: Eraslan et al., 2020). VR technique is also proved to be useful in language learning (Özgün and Sadik, 2003). Students benefited from interactive environment provided by VR setting and were motivated in learning language. Both typically developing controls and atypically developing populations enhance their cognitive and language abilities through advanced technology. The relationship of using VR technique and language learning is tightly close. Although limitations were shown in the virtual supermarket navigation task, such as fewer categories compared to those listed in real life, studies have reported that the virtual supermarket navigation task can assess the abilities of populations with cognitive impairment. A survey of studies with VR intervention on patients with dementia, Alzheimer’s disease, and mild cognitive impairment (MCI) reported the training effectiveness in rehabilitation (Kim et al., 2019). The findings showed that the effect sizes of the interventions on cognition (i.e., memory, attention, concentration, orientation, recall, and wording) were the largest compared to physical fitness, emotion (i.e., anxiety, psychological well-being, and depression), feasibility (i.e., facilitators or barriers to the intervention effect), and execution (i.e., verbal response and performance errors), indicating that VR technology is a promising tool for cognitive rehabilitation. By using the technique, cognitive abilities of people with WS could be enhanced.

The virtual navigation task is suitable for assessing the memory ability of people with neurodegenerative disorders (Yan et al., 2021; Ferreira-Brito et al., 2020). People with MCI were worse at performing virtual navigation tasks than those with TD, but they showed an intervention effect after using VR technology and stopped deterioration. The VR technique is a dynamic tool that is close to daily life and is useful in assessing the abilities of people with neurodevelopmental disabilities. VR is not aimed at rehabilitation or training at the beginning of invention, but later shows an intervention effect after applying it to patients. The aim of this study was to assess the memory ability of people with WS related to semantic knowledge using a virtual navigation task in a supermarket setting and to examine the potential of rehabilitating verbal long-term memory in this population. To assess abilities of people with WS, repetitions of navigation trials were required. VR technique involves many aspects in cognition and language, such as attention, planning, executive function, and memory. By using VR technique, all aspects are involved more or less rather than just one aspect per se. In this study, 14 indices were measured from using VR technique in navigation settings on people with WS and the TD groups. It was hypothesized that people with WS would be worse in these evaluative measurements but would still be observed potentials of rehabilitation through practices by using VR technique. The major goal of this study was to assess the abilities of semantic memory of people with WS; the minor goal was to obtain rehabilitation potentials on people with WS with advanced technology.

## 2 Method

### 2.1 Participants

Twenty people with WS diagnosed at various ages in hospitals took place in this study. The clinical participants were recruited from one of the parents’ groups through a recruitment poster in China. The control groups were recruited from Hunan University and Dongkou Wenchang Street Second Primary School in Hunan Province. All the participants and their caregivers signed a consent form before participating in the study. The mental ages of participants with WS were estimated by the standard Wechsler Scale of Intelligence for Children (WISC-IV). TD groups of the CA- and MA-matched controls were matched individually in gender and age. All the participants were right-handed native Chinese speakers. No differences in ages emerged between people with WS and TD controls. Table 1 presents background information of the participants in each group. This study was approved by the Institutional Review Board of School of Foreign Languages at Hunan University (number 20210628000002).

**Table 1:** Background information of participants.

Tasks	Groups	*N* (F:M)	CA	CA Range (SD)	MA	MA Range (SD)
Virtual supermarket navigation task	College	20 (10:10)	20.2	18.5–22.4 (1.2)	–	–
	5th Grade	20 (10:10)	10.3	9.2–10.11 (0.5)	–	–
	CA	20 (7:13)	12.5	9.5–18.9 (2.7)	–	–
	MA	20 (7:13)	8.8	6.6–15.2 (2.0)	–	–
	WS	20 (7:13)	12.5	9.6–19.2 (2.6)	8.9	6.2–15.2 (2.0)
Familiarity task	5th Grade	20 (10:10)	10.1	9.1–10.11 (0.4)	–	–
Naming and semantic feature judgment task	College	20 (10:10)	20.5	18.11–24.7 (1.5)	–	–

CA: chronological age-matched controls; MA: mental age-matched controls; WS: people with Williams syndrome; F: female; M: male; SD: standard deviation.

### 2.2 Materials and experimental design

**Figure 1:**
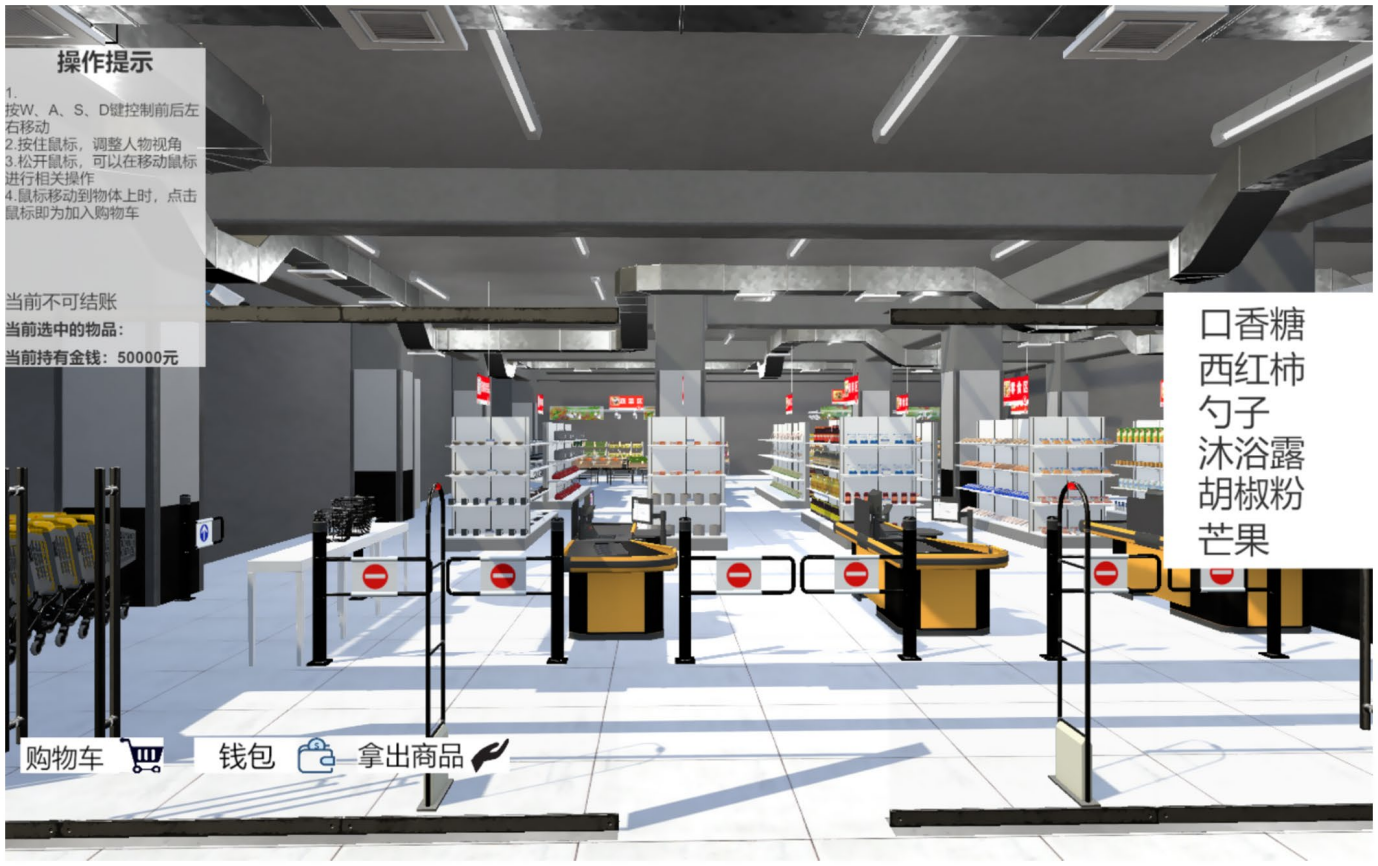
A screen shot of the virtual supermarket navigation task.

This study took the virtual navigation in supermarkets as a paradigm. Four versions (A, B, C, and D) of supermarkets were self-developed and not commercial, which were tested on all participants. Each version was distinct in terms of its layout. The layouts were parallel to medium supermarkets in China. Seven common high-frequency types purchased in supermarkets were displayed, including drinks, snacks, daily necessities, seasoning, kitchen utensils, fruits, and vegetables. Fifteen items tagged with the price for each type were listed. Hence, one hundred and five different items could be shopped in a navigation task. Three automated cashier counters and shopping carts were set up at the entrance. No customers appeared. These navigation tasks could be replicated by using similar designs. An original screenshot of the virtual navigation task in Chinese is shown in Figure 1. The experimenter instructed the participants orally using printed keywords on the left side of the screen.

Participants controlled a shopping cart by clicking the mouse and pressing buttons W, A, S, and D to stand forward, backward, left, or right with stickers on the keyboard as reminders. Participants selected and placed an item in a shopping cart by pressing the left button of the mouse. If a correction was necessary, such as returning an unwanted item, participants could press the right button of the mouse. Participants had to complete the shopping task by selecting all items listed for each navigation and paying the items by clicking the icon of the purse on the screen at the bottom-left corner. The shopping quota for each purchase was a maximum of 50,000 dollars in the account. Three navigation tasks, with different shopping lists provided for each supermarket, were required to complete the study. None of the repeated items were included. All participants could operate the software with their fingers pressing the corresponding buttons and mouse to navigate the designed setting without problems, including people with WS. No motor issues were involved in the tasks performed by people with WS.

All items in the supermarkets were highly familiar to all the participants in the rating task. The Likert rating scale of 5 points from highest 5 to lowest 1. Twenty 5th graders (10F/10M, mean CA = 10.1, SD = 0.4, range = 9.1–10.11) rated all types of items on the shopping lists with the results of 4.94 (SD = 0.07) for fruits, 4.87 (SD = 0.07) for vegetables, 4.88 (SD = 0.07) for snacks, 4.53 (SD = 0.27) for seasoning, 4.89 (SD = 0.13) for kitchen utensils, and 4.98 (SD = 0.05) for daily necessities. All participants received 12 shopping lists to complete the task. Six items, one from each type, were included on each shopping list. Seventy-two items were used as the experimental stimuli.

To ensure that all items were clear in shape as real ones, a generation task to name all items was conducted (college students, n = 20, CA = 20.5, range = 18.11–24.7, SD = 1.5). On the sheets printed with the images excerpted from the software, participants wrote down the names of the items displayed in the supermarkets. The results revealed high consistency in naming the images from the participants. Ninety-four percent of the items were perceived to be similar to a single name. Only a very small percentage of the items had more than one name. This rating suggests that all items listed in supermarkets are similar to the real ones.

### 2.3 Design of VR software tool

The virtual navigation software was generated with 3D Studio Max and 3D Unity in the interactive mode. This software should equip a computer with parameters of R5-4600U CPU, 16GB memory, 512 GB storage capacity, and a 13.3-inch touchscreen. Any goggle or handle was not necessary. A computer mouse and keyboard are required in operation.

### 2.4 Procedure

Upon starting the software, participants were instructed to navigate a supermarket to complete a task by shopping for items on a list. Participants were also instructed to perform all operations, such as returning unwanted items and paying. If the participant could not read, a recording by a male (CA = 28.2, 44.1 kHz, Praat) was presented. The experiment began after practice. This software was non-immersive. Each participant controlled the cart virtually. Participants navigated to locate and place the target items in the shopping cart; then, participants took the items to a cashier and paid for the purchases. There were no repetitions of shopped items. Neither the number nor the temporal sequence of the shopped items mattered because all target items were printed on the computer screen through all the navigations. No time limit was set.

### 2.5 Indices measured in navigation

**Figure 2:**
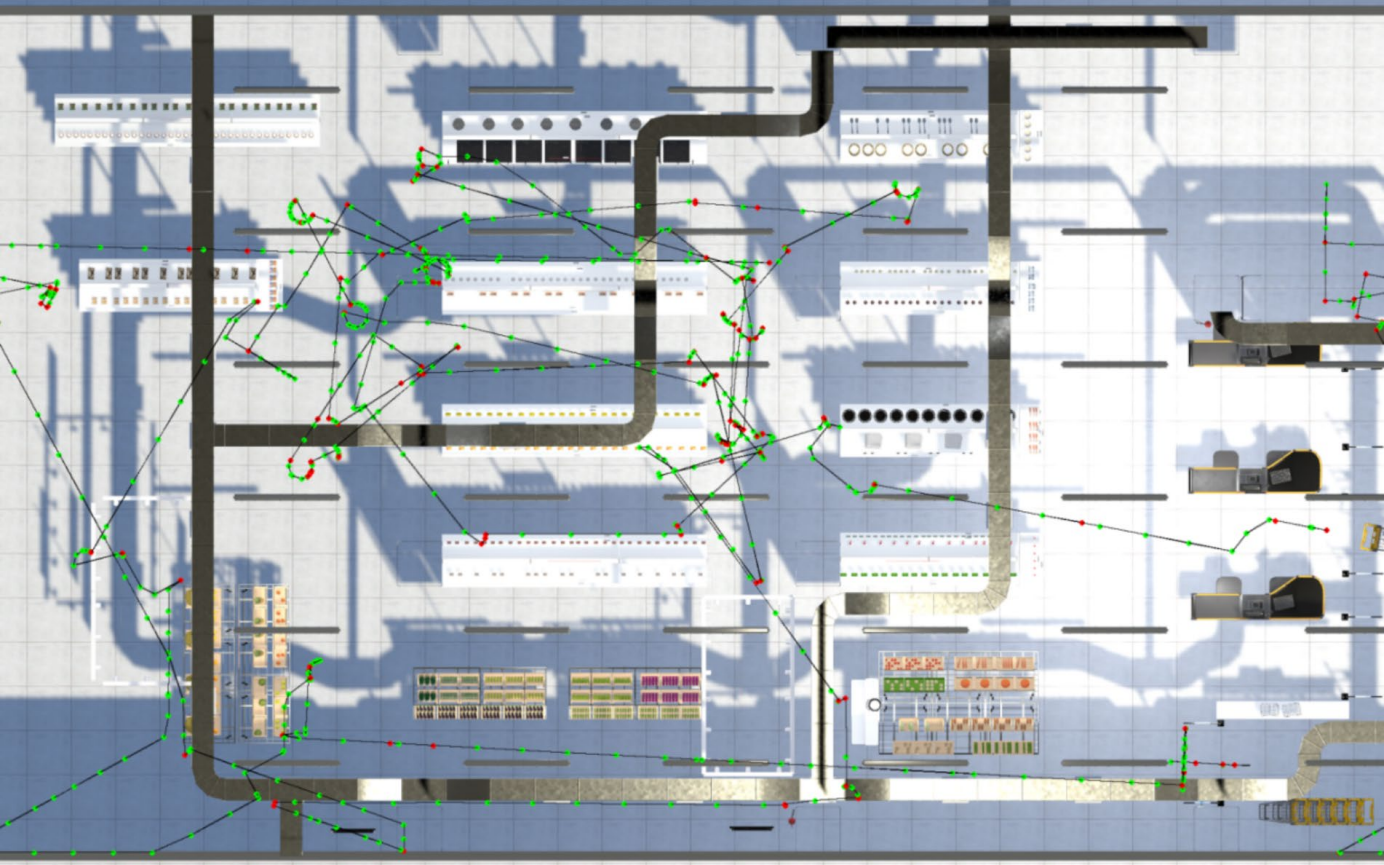
A shopping path of a participant of WS.

**Figure 3:**
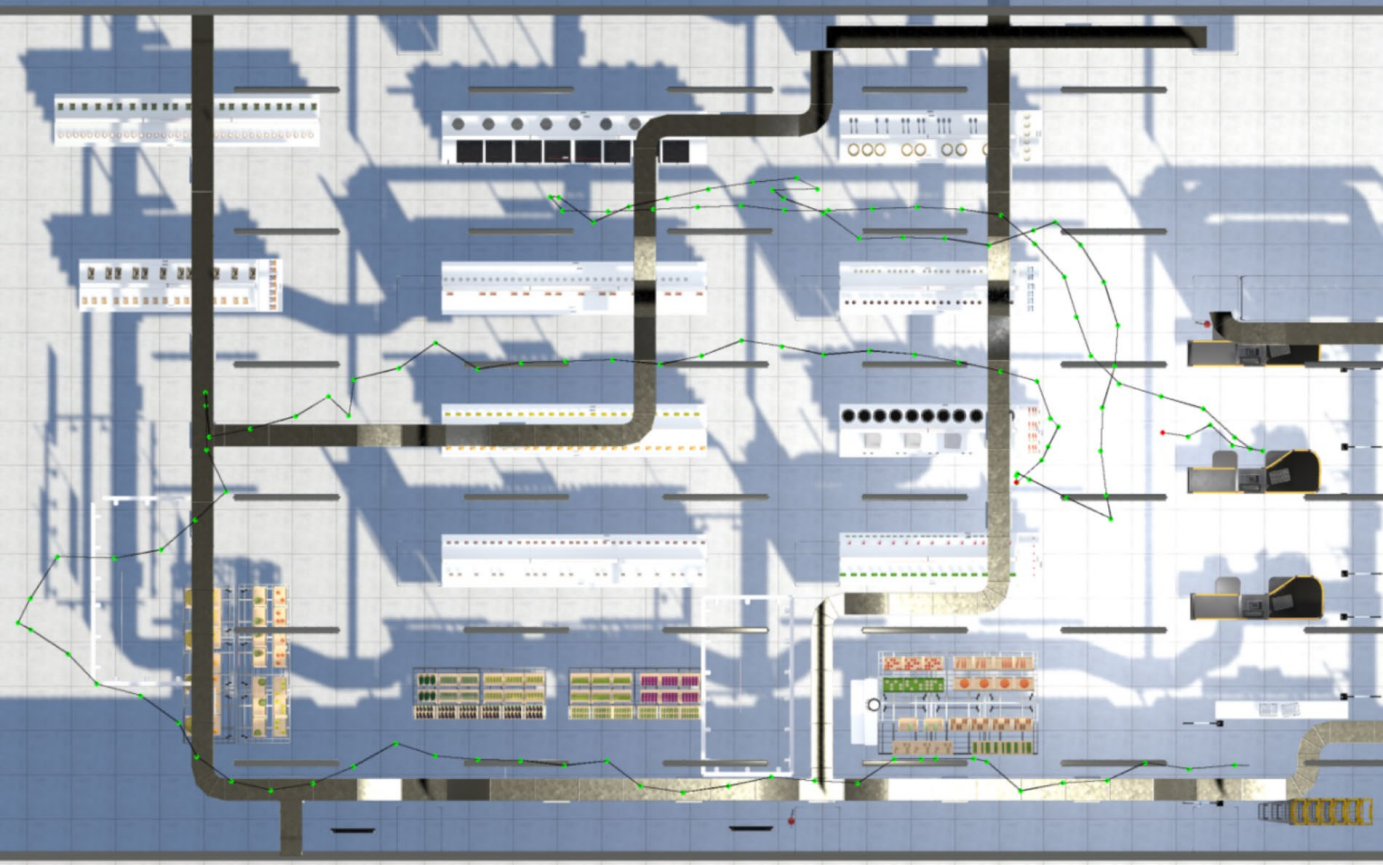
A shopping path of a participant of CA.

Fourteen indices were recorded while the software was operating referenced the studies done by Josman et al. (2008) and Klinger et al. (2009). All indices were related to learning, executive function, planning, and language. These indices were (1) the total length of distance in meters from entrance to exit, (2) the total length of shopping distance in meters from the first item shopped to a cashier counter, (3) the total duration in seconds from entrance to exit, (4) the total shopping duration in seconds from the first item shopped to a cashier counter, (5) the paying duration from the cost displayed on the screen to purchasing icon clicked, (6) the duration of the first item shopped, (7) correct number of types purchased, (8) correct number of items purchased, (9) incorrect number of types purchased, (10) incorrect number of items purchased, (11) pauses during shopping with 2 s as an interval, (12) the total duration of pauses, (13) number of incorrect actions in operating the software while (a) entering a supermarket without clicking the shopping cart, (b) leaving a supermarket without buying, (c) leaving a supermarket without paying, (d) upon paying without clicking the icon of the purse, (e) wondering around after paying, and (14) warning reminders of errors in every 30 s. Figures 2, 3 display the shopping paths of the standing positions of a participant from the WS and CA groups, respectively, every 2 s (with green dots corresponding to the recorded positions and red dots corresponding to the pauses in navigation).

## 3 Results

Data recorded from 14 indices across the participants were collected. The patterns of typically developing (TD) controls from the college students (CS), 5th graders, and CA groups were analyzed first, and data from the CA, MA, and WS groups were compared.

### 3.1 Results of the CS, 5th graders, and CA group

Before looking at the details of analyses, a summary was depicted. Based on the analyses, the results revealed developmental differences in virtual navigation from childhood to adulthood. The 5th graders were less mature than the CS in completing the tasks. The CA controls were generally similar to those of 5th graders, except for a higher number of incorrect actions in operating the software. The statistical results for each group with the mean, standard deviation, and *F* values are listed in Table 2.

**Table 2:** The 14 indices of virtual navigation task from the CS, 5th graders, CA, MA, and WS groups.

No.	Index	College	5th Grade	CA	MA	WS	Group difference at *p-*value^a^	
							College, 5th Grade, CA	CA, MA, WS
1	The total length of distance in meters from entrance to exit	128.8 (21.64)	179.38 (33.42)	171.44 (32.36)	190.88 (31.58)	215.73 (46.82)	16.864, *p* < 0.001	6.982, *p* = 0.002
2	The total length of shopping distance in meters from the first item selected to a cashier counter	115.16 (20.38)	161.33 (29.82)	153.52 (28.22)	169.91 (28.15)	189.46 (43.40)	17.434, *p* < 0.001	5.596, *p* = 0.006
3	The total shopping duration in seconds from entrance to exit	219.54 (43.73)	364.4 (97.18)	325.97 (93.89)	420.22 (118.55)	551.76 (114.83)	16.75, *p* < 0.001	21.403, *p* < 0.001
4	The total shopping duration from the first item selected to a cashier counter	194.71 (39.93)	329.66 (86.18)	293.44 (85.72)	377.36 (103.35)	490.43 (106.03)	17.882, *p* < 0.001	20.03, *p* < 0.001
5	The payment duration from the cost displayed on the screen to the purchasing icon clicked	2.09 (1.19)	6.29 (8.84)	4.96 (6.51)	5.64 (6.25)	5.63 (3.46)	2.256, *p* = 0.114	0.097, *p* = 0.908
6	The duration of the first item shopped upon entrance	24.83 (5.29)	34.74 (13.43)	32.53 (9.85)	42.74 (18.46)	61.33 (15.77)	5.317, *p* = 0.008	18.627, *p* < 0.001
7	Correct number of types purchased	5.92 (0.06)	5.83 (0.12)	5.75 (0.30)	5.47 (0.44)	4.87 (0.86)	3.974, *p* = 0.024	11.664, *p* < 0.001
8	Correct number of items purchased	5.96 (0.11)	5.88 (0.16)	5.81 (0.32)	5.56 (0.48)	4.97 (0.89)	2.457, *p* = 0.095	9.986, *p* < 0.001
9	Incorrect number of types purchased	0.15 (0.12)	0.35 (0.20)	0.31 (0.30)	0.7 (0.37)	1.15 (0.61)	4.45, *p* = 0.016	17.765, *p* < 0.001
10	Incorrect number of items purchased	0.15 (0.12)	0.35 (0.21)	0.31 (0.30)	0.71 (0.38)	1.18 (0.61)	4.485, *p* = 0.016	18.782, *p* < 0.001
11	Pauses during shopping	12.4 (4.94)	26.97 (12.46)	22.7 (11.52)	33.32 (14.23)	42.77 (15.51)	10.771, *p* < 0.001	10.506, *p* < 0.001
12	The total duration of pauses	26.59 (16.95)	97.73 (58.08)	79.5 (50.12)	145.65 (85.13)	216.91 (101.30)	13.271, *p* < 0.001	14.151, *p* < 0.001
13	Number of incorrect actions	0.51 (0.44)	1.02 (0.62)	1.53 (1.07)	1.55 (0.76)	1.84 (0.78)	8.999, *p* < 0.001	0.769, *p* = 0.468
14	Warning reminders of errors	0.57 (0.48)	1.18 (0.80)	1.67 (1.20)	1.7 (0.80)	2.11 (1.04)	7.859, *p* < 0.001	1.122, *p* = 0.333

a: *F*(2,57) for main effect of groups; CA: chronological age-matched group; MA: mental age-matched group; WS: Williams syndrome.

In the analysis of the first index, a one-way ANOVA with the total length of distance in meters from entrance to exit was examined across groups. The results revealed significance [*F*(2,57) = 16.86, *p* < 0.001, η_p_^2^ = 0.37], suggesting that the simple main effect was the difference between the CS (128, SD = 21) and CA controls (171, SD = 32, *p* < 0.001). The difference between the CS and 5th graders (179, SD = 33, *p* < 0.001) was also significant. There was no difference between the 5th graders and CA controls. The results suggest that the CS took the least walking distance to complete the shopping task compared to the CA controls and 5th graders.

In the analysis of the second index, the total shopping distance in meters from the first item shopped to a cashier counter was examined across groups. The results revealed significance [*F*(2,57) = 17.43, *p* < 0.001, η_p_^2^ = 0.38], suggesting that the simple main effect was due to the difference between the CS (115, SD = 20) and CA group (153, SD = 28, *p* < 0.001) and the difference between the CS and 5th graders (161, SD = 29, *p* < 0.001). No difference between the 5th graders and CA controls emerged. The results indicated that the CS took the least distance from purchasing the first item in the supermarket to exit compared to the 5th graders and CA controls.

In the analysis of the third index, the total shopping duration in seconds from entrance to exit was examined across groups. The results reached significance [*F*(2,57) = 16.75, *p* < 0.001, η_p_^2^ = 0.37], suggesting that the simple main effects were from the difference between the CS (219 s, SD = 43) and CA group (325 s, SD = 93, p < 0.001) and the difference between the CS and 5th graders (364 s, SD = 97, *p* < 0.001). No difference between the 5th graders and CA group emerged. The results indicated that the CS took the least total shopping duration compared to the 5th graders and CA controls.

In the analysis of the fourth index, the total shopping duration in seconds from the first item selected to the cashier counter was examined across groups. The results were significant [*F*(2,57) = 17.88, *p* < 0.001, η_p_^2^ = 0.38], suggesting that the simple main effects were from the difference between the CS (194, SD = 39) and CA group (293, SD = 85, *p* < 0.001), and the difference between the CS and 5th graders (329, SD = 86, *p* < 0.001). No difference between the 5th graders and CA group emerged. The results indicated that the CS took the shortest shopping duration from the first item selected to a cashier counter compared to the 5th graders and CA controls.

In the analysis of the fifth index, the payment duration from the cost displayed on the screen to the clicked purchasing icon was taken as the factor to examine group difference. No interaction was obtained, suggesting that the CA controls and 5th graders took similar payment durations as the CS.

In the analysis of the sixth index, the duration of the first item shopped upon entrance in seconds was examined across groups. The results reached significance [*F*(2,57) = 5.31, *p* = 0.008, η_p_^2^ = 0.15], suggesting that the simple main effect was due to the difference between the CS (24, SD = 5) and CA group (32, SD = 9, *p* = 0.019) and the difference between the CS and 5th graders (34, SD = 13, *p* = 0.003). No difference between the 5th graders and CA controls emerged. The results indicated that the CS took the least shopping duration to purchase the first item than the 5th graders and CA controls.

In the analysis of the seventh index, the correct number of purchased types was examined among the groups. The results were significant [*F*(2,57) = 3.97, *p* = 0.024, η_p_^2^ = 0.12], suggesting that the simple main effect was from the difference between the CS (5.91, SD = 0.06) and CA group (5.75, SD = 0.29, *p* = 0.007). Neither a difference between the CS and 5th graders (5.82, SD = 0.11) nor a difference between the CA controls and 5th graders emerged.

In the analysis of the eighth index, the correct number of items purchased was taken as a factor to examine group difference. No interaction was observed, suggesting that the three groups purchased similar numbers of correct items.

An incorrect number of purchase types was examined in the analysis of the ninth index among the groups. The results were significant [*F*(2,57) = 4.45, *p* = 0.016, η_p_^2^ = 0.13], suggesting that the simple main effects were due to the difference between the CS (0.15, SD = 0.11) and CA group (0.31, SD = 0.29, *p* = 0.025) and the difference between the CS and 5th graders (0.34, SD = 0.19, *p* = 0.007). There was no difference between the 5th graders and CA controls. The results indicated that the CS shopped the least incorrect types compared to the 5th graders and CA controls.

In the analysis of the tenth index, the incorrect number of items purchased was examined across groups. The results were significant [*F*(2,57) = 4.48, *p* = 0.016, η_p_^2^ = 0.13], suggesting that the simple main effect was from the difference between the CS (0.15, SD = 0.11) and CA group (0.31, SD = 0.29, *p* = 0.026) and the difference between the CS and 5th graders (0.35, SD = 0.20, p = 0.007). No difference between the 5th graders and CA controls emerged. The results indicated that the CS shopped the least incorrect number of items purchased compared to the 5th graders and CA controls.

In the analysis of the eleventh index, pauses during shopping with an interval of 2 s were examined across groups. The results were significant [*F*(2,57) = 10.77, *p* < 0.001, η_p_^2^ = 0.27], suggesting that the simple main effects were due to the difference between the CS (12, SD = 4) and CA group (26, SD = 12, *p* = 0.002) and the difference between the CS and 5th graders (22, SD = 11, *p* < 0.001). No difference between the 5th graders and CA controls emerged. The results indicated that the CS paused least than the 5th graders and CA controls did.

In the analysis of the twelfth index, the total duration of pauses in seconds was examined across groups. The results were significant [*F*(2,57) = 13.27, *p* < 0.001, η_p_^2^ = 0.31], suggesting that the simple main effect was from the difference between the CS (26, SD = 16) and CA group (79, SD = 50, *p* = 0.001), and the difference between the CS and 5th graders (97, SD = 58, *p* < 0.001). No difference between the 5th graders and CA controls emerged. The results indicated that the CS paused the shortest compared to the 5th graders and CA controls.

In the analysis of the thirteenth index, the number of incorrect actions when operating the software was examined across groups. The results reached significance [*F*(2,57) = 8.99, *p* < 0.001, η_p_^2^ = 0.24], suggesting that the simple main effect was due to the differences among the three groups [CS = 0.50, SD = 0.43; CA = 1.52, SD = 1.06; 5th graders, 1.02, SD = 0.62]. The results suggest that the CS made fewer incorrect actions than the 5th graders (*p* = 0.037) and CA controls (*p* < 0.001). The 5th graders made fewer incorrect actions compared to the CA controls (*p* = 0.040). The CA controls made the highest number of incorrect operations among the three groups.

In the analysis of the fourteenth index, warning reminders of errors every 30 s were examined across groups. The results were significant [*F*(2,57) = 7.85, *p* = 0.001, η_p_^2^ = 0.21], suggesting that the simple main effects were due to the difference between the CS (0.57, SD = 0.48) and CA group (1.67, SD = 1.20, *p* < 0.001) and the difference between the CS and 5th graders (1.17, SD = 0.80, *p* = 0.034). No difference between the 5th graders and CA controls emerged. The results indicated that the CS received the least warning reminders of errors compared to the 5th graders and CA controls.

### 3.2 Results of the CA and MA groups and people with WS

A summary was depicted before looking at the details of analysis. Based on the results across groups, atypical processing of the WS group in navigation compared to the CA and MA groups was observed. The MA group differed from the CA group in terms of longer shopping duration from entrance to exit, longer total duration from the first item shopped to a cashier counter, and longer duration of the first item purchased upon entrance. These results suggest that the MA group was still in development. The statistical results for each group with the mean, standard deviation, and *F* values is listed in Table 2.

In the first index, the total distance in meters from entrance to exit was examined across groups. The results were significant [*F* (2,57) = 6.98, *p* = 0.002, η_p_^2^ = 0.19], suggesting that the simple main effect was due to the difference between the WS group and the two control groups [WS, 215 (SD = 46) vs. CA, 171 (SD = 32), *p* < 0.001; WS vs. MA, 190 (SD = 31), *p* = 0.041]. No difference was observed between the CA and MA groups. The results suggest that the WS group shopped the longest distance from entrance to exit compared to the CA and MA groups.

In the second index, the total shopping distance in meters from the first item shopped to a cashier counter was examined across groups. The results were significant [*F*(2,57) = 5.59, *p* = 0.006, η_p_^2^ = 0.16], suggesting that the simple main effect was due to the difference between the WS (189, SD = 43) and CA (153, SD = 28) groups (*p* = 0.001). The MA group (169, SD = 28) did not differ from the other two groups. The results suggest that the WS group walked longest distance from the first item purchased to a cashier counter than the TD controls.

In the third index, the total shopping duration in seconds from entrance to exit was examined across groups. The results were significant [*F*(2,57) = 21.40, *p* < 0.001, η_p_^2^ = 0.42], suggesting that the simple main effect was due to the difference between the WS group and the TD controls [WS, 551 (SD = 114) vs. CA, 325 (SD = 93), *p* < 0.001; WS vs. MA, 420 (SD = 118), *p* < 0.001]. The difference between the CA and MA groups was statistically significant (*p* = 0.009). The results suggest that the WS group shopped longest duration in total compared to the CA and MA groups. Compared with the CA group, the MA group shopped longer in total from the entrance to the exit.

In the fourth index, the total shopping duration from the first item selected to a cashier counter was examined across groups. The results reached significance [*F*(2,57) = 20.03, *p* < 0.001, η_p_^2^ = 0.41], suggesting the simple main effects were from the differences between the WS group and the TD controls [WS, 490 (SD = 106) vs. CA, 293 (SD = 85), *p* < 0.001; WS vs. MA, 377 (SD = 103), *p* = 0.001]. The difference between the CA and MA groups was significant (*p* = 0.009). The results indicated that the WS group shopped longest duration from the first item purchased compared to the CA and MA groups. In turn, the MA group shopped longer in total duration from the first item purchased to a cashier counter than did the CA group.

In the fifth index, the payment duration from the cost displayed on the screen to the clicked purchasing icon was examined across groups. No significance emerged [WS, 5 (SD = 3); CA, 4 (SD = 6); MA, 5 (SD = 6)], implying a similar payment duration from the cost displayed to the purchase icon clicked.

In the sixth index, the duration of the first item shopped upon entrance was examined across groups. The results reached significance [*F*(2,57) = 18.62, *p* < 0.001, η_p_^2^ = 0.39], suggesting the simple main effects were from the differences between the WS group and the TD controls [WS, 61 (SD = 15) vs. CA, 32 (SD = 9), *p* < 0.001; WS vs. MA, 42 (SD = 18), *p* < 0.001]. The difference between the CA and MA groups was statistically significant (*p* = 0.037). The results indicated that the WS group took longer to purchase the first item than the CA and MA groups. The MA group, in turn, shopped longer duration for the first item purchased upon entrance than the CA group.

In the seventh index, the correct number of types purchased was examined across groups. The results reached significance [*F* (2,57) = 11.66, *p* < 0.001, η_p_^2^ = 0.29], suggesting the simple main effects were from the differences between the WS group and the TD controls [WS, 4.87 (SD = 0.86) vs. CA, 5.7 (SD = 0.29), *p* < 0.001; WS vs. MA, 5.47 (SD = 0.44), *p* = 0.002]. No significant difference was observed between the CA and MA groups. The results indicated that the WS group purchased the least number of correct types compared to the CA and MA groups.

In the eighth index, the correct number of items purchased was examined across groups. The results reached significance [*F*(2,57) = 9.98, *p* < 0.001, η_p_^2^ = 0.25], suggesting the simple main effects were from the differences between the WS group and the TD controls [WS, 4.96 (SD = 0.88) vs. CA, 5.80 (SD = 0.31), *p* < 0.001; WS vs. MA, 5.55 (SD = 0.47), *p* = 0.003]. No difference was observed between the CA and MA groups. The results indicated that the WS group purchased the least number of correct items compared to the CA and MA groups.

In the ninth index, the incorrect number of purchased types was examined across groups. The results reached significance [*F*(2,57) = 17.76, *p* < 0.001, η_p_^2^ = 0.38], suggesting the simple main effects were from the differences between the WS group and the TD controls [WS, 1.15 (SD = 0.61) vs. CA, 0.31 (SD = 0.29), *p* < 0.001; WS vs. MA, 0.69 (SD = 0.36), *p* = 0.002]. The difference between the CA and MA groups was significant (*p* = 0.009). The results indicated that the WS group shopped the highest incorrect types compared to the CA and MA groups. The CA group purchased fewer incorrect types than the MA group did.

In the tenth index, an incorrect number of items purchased was examined across groups. The results reached significance [*F* (2,57) = 18.78, *p* < 0.001, η_p_^2^ = 0.39], suggesting the simple main effects were from the differences between the WS group and the TD controls [WS, 1.17 (SD = 0.60) vs. CA, 0.31 (SD = 0.29), *p* < 0.001; WS vs. MA, 0.70 (SD = 0.37), p = 0.002]. The difference between the CA and MA groups was statistically significant (*p* = 0.007). The results indicated that the WS group shopped the highest incorrect items compared to the CA and MA groups. The CA group purchased fewer incorrect items than the MA group did.

In the eleventh index, pauses recorded every 2 s during shopping were examined across groups. The results were significant [*F*(2,57) = 10.50, *p* < 0.001, η_p_^2^ = 0.26], suggesting that the simple main effect was due to the differences between the WS group and the TD controls [WS, 42 (SD = 15) vs. CA, 22 (SD = 11), *p* < 0.001; WS vs. MA, 33 (SD = 14), *p* = 0.035]. The difference between the CA and MA groups was statistically significant (*p* = 0.019). The results indicated that the WS group paused more during shopping than the CA and MA groups did. The CA group paused fewer during shopping than the MA group did.

In the twelfth index, the total duration of pauses was examined across groups. The results were significant [*F*(2,57) = 14.15, *p* < 0.001, η_p_^2^ = 0.33], suggesting that the simple main effect was due to the differences between the WS group and the TD controls [WS, 216 (SD = 101) vs. CA, 79 (SD = 50), *p* < 0.001; WS vs. MA, 145 (SD = 85), *p* = 0.008]. The difference between the CA and MA groups was statistically significant (*p* = 0.013). The results indicated that the WS group paused the longest during shopping compared to the CA and MA groups. The CA group paused shorter compared to the MA group.

In the thirteenth index, the number of incorrect actions in operating the software was examined across groups. No significant differences emerged [WS, 1.83 (SD = 0.78); CA, 1.52 (SD = 1.06); MA, 1.55 (SD = 0.75]). The WS group performed a similar number of incorrect actions as TD controls.

In the fourteenth index, warning reminders of errors displayed were examined across groups. No significance emerged [WS, 2.10 (SD = 1.04); CA, 1.67 (SD = 1.20); MA, 1.70 (SD = 0.79)], suggesting the WS group received similar number of warning reminders of errors as TD controls.

## 4 Error patterns of all groups

To investigate the error patterns of all groups, the incorrect number of types shopped and the incorrect number of items purchased were compared. Five error types were analyzed: (1) incorrect items purchased under correct types, (2) extra incorrect items purchased under correct types, (3) extra number of target items purchased, (4) missed correct items, and (5) incorrect types purchased. The data collected from the CS, 5th graders, and CA groups were compared first; the data from the CA, MA, and WS groups were then compared. These five error types were taken as the within-participants factor, and groups as the between-participants factor in a two-way analysis of variance with repeated measures.

### 4.1 Error patterns of the CS, 5th graders, and CA group

The results revealed an interaction between the five error types and groups [*F*(8,228) = 2.10, *p* = 0.037, η_p_^2^ = 0.06]. The simple main effect was from the group difference on extra items purchased under correct types [*F*(2,57) = 3.60, *p* = 0.034, η_p_^2^ = 0.11] and correct items missed [*F*(2,57) = 5.30, *p* = 0.008, η_p_^2^ = 0.15]. Among the extra items purchased under correct types, the CS purchased the fewest extra items under correct types (0.80, SD = 1.05) compared to the 5th graders (1.95, SD = 1.66) and the CA group (1.45, SD = 1.47). Only the CS and 5th graders reached statistical significance (*p* = 0.01). In the correct items missed, the CS missed the fewest correct items (0.20, SD = 0.41) compared to the 5th graders (0.30, SD = 0.57) and the CA group (1.05, SD = 1.39). The differences between the CA group and CS (*p* = 0.004) and the 5th graders (*p* = 0.011) were significant. The CS made the fewest errors among the groups. The 5th graders behaved like the CS regarding missing target items; the CA controls missed the highest correct items.

Another simple main effect was from type effect in each group [CS, *F*(4,76) = 3.54, *p* = 0.01, η_p_^2^ = 0.15; 5th graders, *F*(4,76) = 12.21, *p* < 0.001, η_p_^2^ = 0.31; CA, *F*(4,76) = 5.34, *p* = 0.001, η_p_^2^ = 0.22]. In the CS group, both missed correct items (0.20, SD = 0.41) and incorrect types purchased (0.20, SD = 0.41) significantly erred less than incorrect items purchased under correct types (0.80, SD = 0.61) and extra items purchased under correct types (0.80, SD = 1.05). The latter two error types were erred more than the former two in the CS. The error of the extra correct items purchased yielded no difference between groups.

The 5th graders differed from the CS in two error types. The incorrect items purchased under correct types (1.80, SD = 1.32) and extra items purchased under correct types (1.95, SD = 1.66, *p* = 0.013) were significantly higher than those of extra correct items purchased (0.65, SD = 0.93, *p* = 0.001) in the 5th graders. The 5th graders differed from the CA group in three comparisons. The 5th graders showed differences between incorrect items purchased under correct types (1.8, SD = 1.32) and missed correct items (0.30, SD = 0.57), between extra items purchased under correct types (1.95, SD = 1.66) and missed correct items, and between incorrect items purchased under correct types and extra correct items purchased (0.65, SD = 0.93). The CA did not show these patterns. The CA group showed differences between extra items purchased under correct types (1.45, SD = 1.27) and extra target items purchased (0.70, SD = 0.86, *p* = 0.036), and between missed correct items (1.05, SD = 1.39) and incorrect types purchased (0.15, SD = 0.36, *p* = 0.009). Unlike the CS, no differences in the CA group emerged between incorrect items purchased under correct types and missed correct items and between extra items purchased under correct types and missed correct items. These findings suggest that the CA group missed more target items compared to the CS and 5th graders. In sum, the three TD groups exhibited different error patterns in virtual navigation.

**Figure 4:**
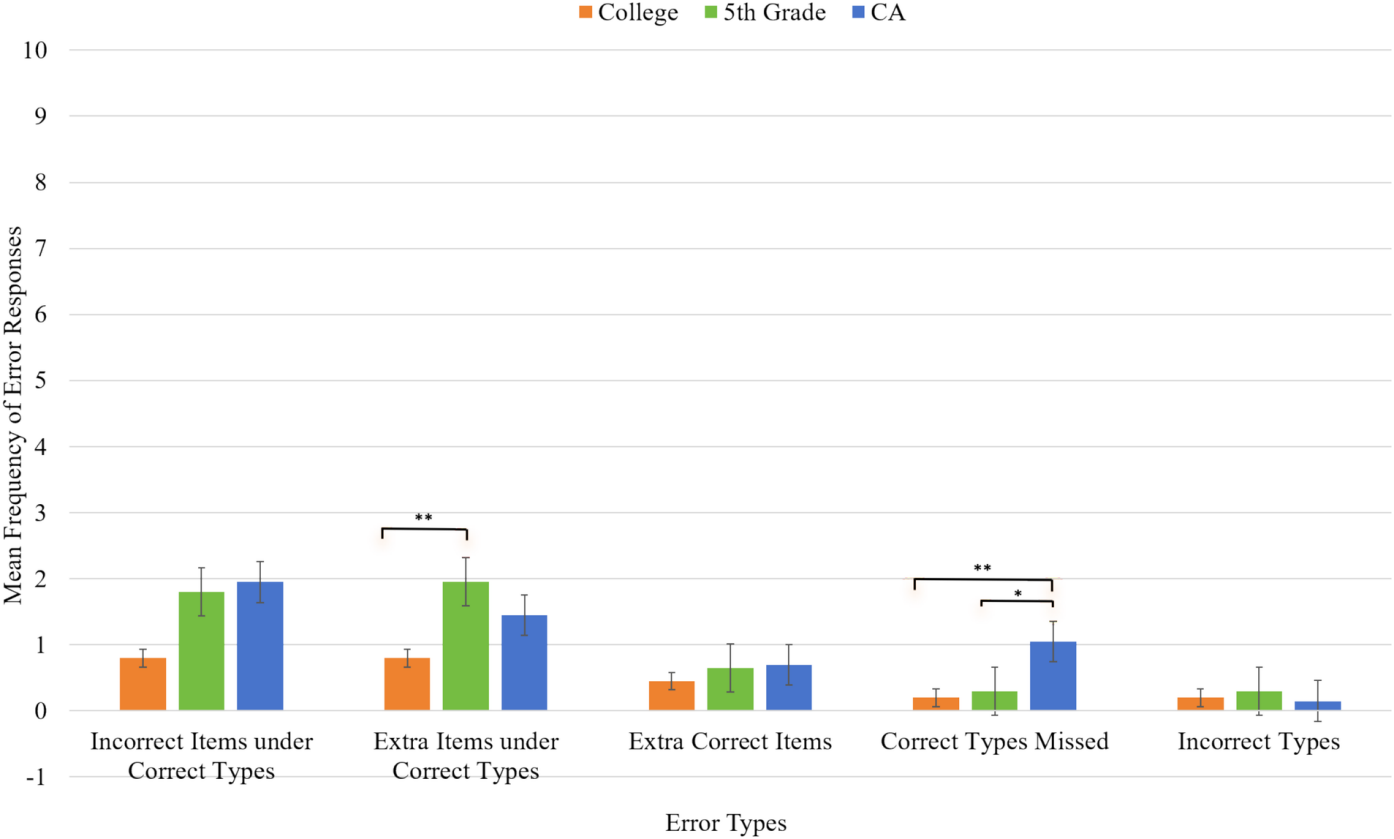
Error patterns in percentage of the college students, 5th graders, and CA group. *p<0.05, **p<0.01.

The main effect of group was significant [*F*(2,57) = 5.07, p = 0.009, η_p_^2^ = 0.15], suggesting fewer errors were made in the CS (0.49, SE = 0.13) than in the 5th graders (1, SE = 0.13) and the CA group (1, SE = 0.13). The latter two groups were not statistically significant. The main effect of error type was significant [*F*(4,228) = 17.64, *p* < 0.001, η_p_^2^ = 0.23]. The incorrect types purchased (0.21, SE = 0.05) were responded with the fewest errors among all error types. Incorrect items purchased under correct types (1.51, SE = 0.21) and extra items purchased under correct types (1.40, SE = 0.17) were erred more than extra correct items purchased (0.60, SE = 0.11), missed correct items (0.51, SE = 0.11), or incorrect types purchased. The extra correct items purchased and missed correct items were erred less than those in other three types. A graph depicting the differences among groups across each error type is shown in Figure 4.

### 4.2 Error patterns of the CA, MA, and WS group

**Figure 5:**
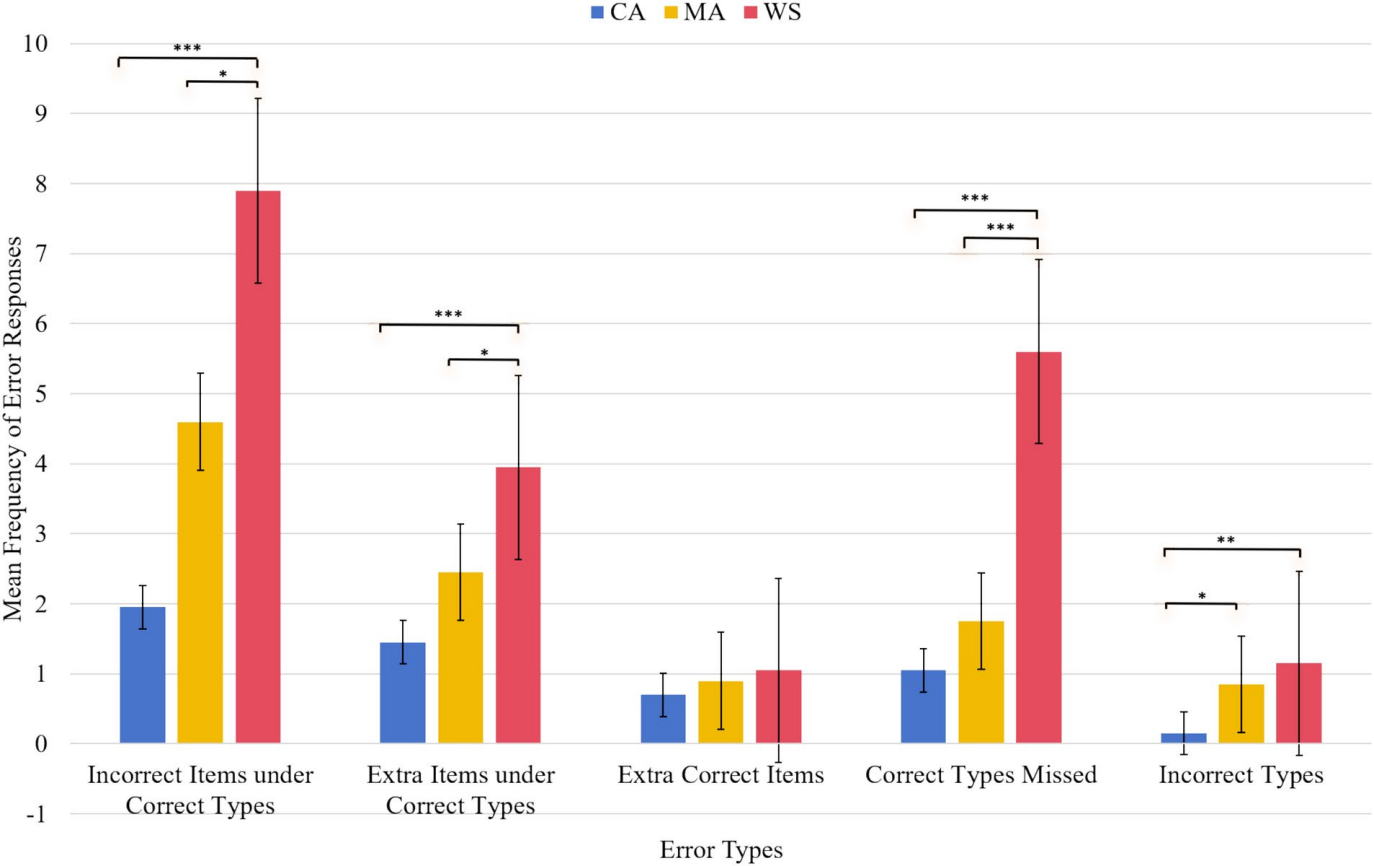
Error patterns in percentage of the CA, MA, and WS group. *p<0.05, **p<0.01, ***p<0.001.

A summary was depicted before examining the details. The WS group showed the highest errors compared to the CA and MA groups for all indices (except for the extra target items purchased). Among the differences, the WS group showed deviant patterns to incorrect items purchased under correct types, extra items purchased under correct types, and missed correct items together with a delayed pattern to incorrect types purchased. The MA group showed CA-like development to incorrect items purchased under correct types, extra items purchased under correct types, and correct items missed, but differed from the CA group to incorrect types purchased. A graph depicting the differences among groups across each error type is shown in Figure 5.

The results revealed an interaction between the five error types and the groups [*F*(8,228) = 5.43, *p* < 0.001, η_p_^2^ = 0.16]. The simple main effect was from the group difference on incorrect items purchased under correct types [*F*(2,57) = 10.09, *p* < 0.001, η_p_^2^ = 0.26], extra items purchased under correct types [*F*(2,57) = 8.32, *p* = 0.001, η_p_^2^ = 0.22], missed correct items [*F*(2,57) = 10.94, *p* < 0.001, η_p_^2^ = 0.27], and incorrect types purchased [*F*(2,57) = 4.71, *p* = 0.013, η_p_^2^ = 0.14]. For the incorrect items purchased under correct types, the WS group (7.90, SD = 5.73) scored more than the MA (4.60, SD = 3.69, *p* = 0.016) and CA groups (1.95, SD = 2.52, *p* < 0.001). No significant difference emerged between the MA and CA groups. For the extra items purchased under correct types, the WS group (3.95, SD = 2.21) erred more than the MA group (2.45, SD = 2.21, *p* = 0.018) and the CA group (1.45, SD = 1.27, *p* < 0.001). No differences emerged between the MA and CA groups. For missed correct items, the WS group (5.60, SD = 5.03) scored more than the MA group (1.75, SD = 2.38, *p* = 0.001) and the CA group (1.05, SD = 1.39, *p* < 0.001). No difference emerged between the MA and CA groups. For the incorrect types purchased, the WS group (1.15, SD = 1.49) erred more than the MA group (0.85, SD = 0.98) and the CA group (0.15, SD = 0.36, *p* = 0.004). No difference was observed between the WS and MA groups. A significant difference emerged between the MA and CA groups (*p* = 0.041). There was no difference among the groups in terms of the extra correct items purchased.

Another simple main effect was from type effect in each group [CA, *F*(4,76) = 5.34, p < 0.001, η_p_^2^ = 0.22; MA, *F*(4,76) = 9.85, *p* < 0.001, η_p_^2^ = 0.34; WS, *F*(4,76) = 16.33, *p* < 0.001, η_p_^2^ = 0.46]. In the CA group, errors made in the incorrect types purchased (0.15, SD = 0.36) were the lowest among all error types [incorrect items purchased under correct types (1.95, SD = 2.52), extra items purchased under correct types (1.45, SD = 1.27), extra target items purchased (0.70, SD = 0.86), and correct items missed (1.05, SD = 1.39]). Moreover, the CA group purchased more extra items under correct types than extra target items (*p* = 0.036).

In the MA group, the errors made for the incorrect types purchased were significantly lower (0.85, SD = 0.22) compared to the errors made for the incorrect items purchased under correct types (4.60, SD = 0.82, p < 0.001) and the extra items purchased under correct types (2.45, SD = 0.49, *p* = 0.001). The MA group made more errors in the incorrect items purchased under correct types (4.60, SD = 0.82) than in the missed correct items (1.75, SD = 0.53). The MA group purchased more correct items than incorrect items under correct types, and extra items purchased under correct types. Moreover, differences in the error patterns between the MA and CA groups emerged in five comparisons. While the CA group showed differences between the errors in the incorrect types purchased, the errors in the extra correct items purchased, and the errors in the missed correct items, the MA group did not show these patterns. The CA group showed differences between the errors in the incorrect items purchased under correct types and the errors in the extra correct items purchased and the errors in the missed correct items, whereas the MA group did not show these patterns. In contrast, while the MA group showed a difference between the errors in the missed correct items and the errors in the incorrect types purchased, the CA group did not show these patterns.

In the WS group, the errors made to the incorrect types purchased (1.15, SD = 0.33) were the least compared to the errors made to the incorrect items purchased under correct types (7.90, SD = 1.28), errors in the extra items purchased under the correct types (3.95, SD = 0.49), and errors made to the missed correct items (5.60, SD = 1.15). The WS group erred less to missed correct items than to incorrect items purchased under correct types, but more to errors in extra target items purchased. The WS group made the least errors to the extra correct items purchased (1.05, SD = 0.24) compared to the errors to the incorrect items purchased under correct types and the errors to the extra items purchased under correct types. The WS group erred more incorrect items purchased under correct types than extra items purchased under correct types. Furthermore, differences between the WS and MA groups emerged in all three comparisons. While the WS group showed differences between the errors to the missed correct items and the errors to the extra correct items purchased, and between the errors to the missed correct items and the error to the incorrect types purchased, the WS group did not show these patterns. The MA group showed a difference between the errors in the incorrect items purchased under correct types and the errors in the extra items purchased under correct types. Moreover, differences between the WS and CA groups emerged in five comparisons. While the WS group showed differences between the errors in the incorrect items purchased under correct types and the errors in the extra items purchased under correct types, the errors in the extra target items purchased, and the errors in the missed correct items, the CA group did not show these patterns. The WS group showed a difference between the errors in the extra correct items purchased and those in the missed correct items, whereas the CA group did not show this pattern. In contrast, while the CA group showed a difference between the errors in the extra correct items purchased and the errors in the incorrect types purchased, the WS group did not show this pattern.

The WS group (3.93, SE = 0.34) made significantly more errors than the CA (1.06, SE = 0.34, *p* < 0.001) and MA (2.11, SE = 0.34, *p* < 0.001) groups [group main effect, *F*(2,57) = 17.96, *p* < 0.001, η_p_^2^ = 0.38]. The latter two groups differed significantly (*p* = 0.034). The main effect of type was significant [*F*(4,228) = 29.28, *p* < 0.001, η_p_^2^ = 0.339]. The errors for the incorrect types purchased (0.71, SD = 1.36) were the lowest among all the error types. Errors to the extra items purchased under correct types (2.61, SE = 0.25) and errors to the missed correct items (2.80, SE = 0.42) showed significant differences in the errors made to the incorrect items purchased under correct types (4.81, SE = 0.54), errors in the extra correct items purchased (0.88, SE = 0.13), and errors to the incorrect types purchased (0.71, SE = 0.13). The errors made to the extra correct items purchased and the errors to the incorrect types purchased were significantly different from the errors to the incorrect items purchased under the correct types, the errors to the extra items purchased under the correct types, and the errors to the missed correct items.

## 5 Comparisons of strategies of replacement, confusion, missing, extra-buying, and incorrect purchasing across groups

To determine whether participants showed domain-specific impairments, errors in each category were analyzed based on five possible strategies: (1) replacement of the target items (i.e., participants purchased items other than the target items with other items under correct type), (2) confusion of the target items (i.e., participants not only purchased the target item but also another non-target item under correct type), (3) missed correct items (i.e., participants checked out the purchased items without buying the target items on the shopping lists), (4) extra buying of the target items (i.e., participants purchased more than one target item under correct type without replacement), and (5) incorrect purchasing of the target items (i.e., participants purchased items under incorrect types). Two-way analyses of variance were conducted with categories as the within-participants factor and groups as the between-participants factor.

A summary was depicted before examining the details of analyses. The WS group erred most regardless of the strategy of replacement, confusion together with the phenomena of correct items missed and incorrect type purchased among groups. Compared to the CA and MA groups, the WS group confused vegetables more, replaced the categories of kitchen utensils and daily necessities more, missed target items more, and purchased the incorrect type (i.e., drink) more. These findings indicate that the WS group completed the task in atypical way from TD controls.

In the analyses of replacement strategies for TD controls, no significant interaction between type and groups emerged. This finding suggests that the control groups made similar errors across categories, with most unfamiliarity with seasoning and familiarity with vegetables. The CS erred less (0.13, SE = 0.06) than the 5th graders (0.30, SE = 0.06), who in turn erred less than the CA group (0.35, SE = 0.06); however, no significant difference emerged among the groups. Only the difference in types was significant [*F*(5,285) = 12.71, *p* < 0.001, η_p_^2^ = 0.18]. The category of vegetables ranked least (0.033, SE = 0.024) in errors compared to the other categories (except for the category of kitchen utensils) [vegetables vs. fruits, 2.33, SE = 0.089, *p* = 0.038; vegetables vs. snacks, 0.183, SE = 0.064, *p* = 0.037; vegetables vs. seasoning, 0.767, SE = 0.130, *p* < 0.001; vegetables vs. daily necessities, 0.217, SE = 0.058, *p* = 0.007]. Seasoning were erred more frequently than fruits, vegetables, snakes, kitchen utensils, and daily necessities (all at *p* < 0.001).

In the analyses of the replacement strategy in the WS group and TD controls of the MA and CA matched groups, no interaction emerged. The WS group made more replacement errors (1.38, SE = 0.16) than the MA (0.81, SE = 0.16) and CA (0.35, SE = 0.16) groups [main effect of group, *F*(2,57) = 9.64, *p* < 0.001, η_p_^2^ = 0.25]. No difference was observed between the CA and MA groups. The main effect of type was significant [*F*(5,285) = 11.75, *p* < 0.001, η_p_^2^ = 0.17]. The category of seasoning was erred most (1.73, SE = 0.21) compared to the other categories (*p* < 0.001 [seasoning vs. fruits, 0.65, SE = 0.13; seasoning vs. vegetables, 0.73, SE = 0.13; seasoning vs. snacks, 0.65, SE = 0.16; seasoning vs. kitchen utensils, 0.53, SE = 0.11; seasoning vs. daily necessities, 0.80, SE = 0.12]). The category of kitchen utensils was erred least among all categories, but only the difference between daily necessities was significant (*p* = 0.034). In sum, the WS group made more replacement errors than the MA and CA groups; the CA group made replacement errors similar to those of the 5th graders and CS.

In the analyses of confusion strategies in TD controls, neither the interaction nor main effect of type was significant. Only the main effect of group was significant [*F*(2,57) = 3.87, *p* = 0.026, η_p_^2^ = 0.12], suggesting that the 5th graders showed more confusion (0.33, SE = 0.051) than the CS (0.13, SE = 0.051, *p* = 0.007) and CA groups (0.24, SE = 0.051, ns).

In the analysis of confusion strategies in the WS group and TD controls of the MA and CA groups, an interaction emerged [*F*(10,285) = 2.00, *p* = 0.033, η_p_^2^ = 0.06]. The simple main effect was the group effect on the categories of fruits [*F*(2,57) = 3.19, *p* = 0.048, η_p_^2^ = 0.10] and vegetables [*F*(2,57) = 6.04, *p* = 0.004, η_p_^2^ = 0.17]. *Post hoc* analyses revealed that the WS group confused more than the CA and MA groups while shopping for fruits and vegetables [fruits: WS, 0.45, SD = 0.68; MA, 0.10, SD = 0.44; CA, 0.10, SD = 0.30; vegetables: WS, 1.35, SD = 1.53; MA, 0.40, SD = 0.68; CA, 0.35, SD = 0.58]. Another simple main effect was the type effect on groups [CA, ns; MA, *F*(5,95) = 2.47, *p* = 0.038, η_p_^2^ = 0.115; WS, *F*(5,95) = 2.89, *p* = 0.018, η_p_^2^ = 0.132]. The results revealed that the MA group confused the categories of seasoning (0.60, SD = 0.82, *p* = 0.038) and daily necessities (0.70, SD = 0.80, *p* = 0.007) compared with fruits (0.10, SD = 0.44). The MA group confused daily necessities more than snacks (0.20, SD = 0.52, p = 0.038). Moreover, the WS group confused vegetables (1.35, SD = 1.53) more than fruits (0.45, SD = 0.68) and daily necessities (0.45, SD = 0.60). The results suggest that the CA group showed little confusion across all categories, but the MA and WS groups showed different patterns of confusion when selecting the target items. In summary, while seasoning and daily necessities confused the MA group more, vegetables confused the WS group more often.

The main effect of group was significant [*F*(2,57) = 9.01, *p* < 0.001, η_p_^2^ = 0.24], suggesting that the WS group (0.70, SE = 0.07) confused more than the MA (0.40, SE = 0.07) and CA (0.24, SE = 0.07) groups. No difference was observed between the CA and MA groups. The main effect of type was significant [*F*(5,285) = 3.40, *p* = 0.005, η_p_^2^ = 0.05]. Fruits were the least confused (0.21, SE = 0.06) compared to vegetables (0.70, SE = 0.13, *p* = 0.004), seasoning (0.56, SE = 0.10, *p* = 0.007), and daily necessities (0.46, SE = 0.08, *p* = 0.017). Snacks (0.36, SE = 0.07) were less confused than vegetables.

In the analysis of the extra-buying strategy on TD controls, neither the interaction nor the group reached significance. Only the main effect of type was significant [*F*(5,285) = 4.10, *p* = 0.001, η_p_^2^ = 0.06], suggesting that kitchen utensils (0) and daily necessities (0.017, SE = 0.017) were erred less than fruits (0.16, SE = 0.05), vegetables (0.18, SE = 0.05), and seasonings (0.16, SE = 0.05). The findings suggest that TD controls selected extra target items for fruits, vegetables, and seasoning categories. In the analyses of the extra-buying strategy on the WS group and TD controls of the MA and CA groups, neither the interaction nor the group reached significance. The main effect of type was significant [*F*(5,285) = 2.75, *p* = 0.019, η_p_^2^ = 0.04]. All participants bought extra target items in vegetables (0.28, SE = 0.06) than seasoning (0.11, SE = 0.04), kitchen utensils (0.05, SE = 0.02), and daily necessities (0.10, SE = 0.04). Moreover, all participants bought more target items in snacks (0.18, SE = 0.05) than in kitchen utensils. These results suggest that the WS and TD groups showed similar patterns of buying extra target items.

In the analyses of the missed target item phenomenon in TD groups, no interaction emerged. The CA group missed more target items (0.17, SE = 0.03) than did the CS group (0.03, SE = 0.03, *p* = 0.004) and 5th graders (0.05, SE = 0.03, *p* = 0.011) [main effect of group, *F*(2,57) = 5.39, *p* = 0.008, η_p_^2^ = 0.15]. No difference emerged between the CS and 5th graders. Fruits were purchased without missing (0) and were significantly different from the categories of seasoning (0.15, SE = 0.06, *p* = 0.020), kitchen utensils (0.13, SE = 0.05, *p* = 0.014), and daily necessities (0.13, SE = 0.04, *p* = 0.002). In the analyses of missed target item phenomenon in the WS group and TD controls of the MA and CA groups, neither interaction nor type effect was significant. The WS group missed more target items (0.93, SE = 0.12) than the CA (0.17, SE = 0.12, *p* < 0.001) and MA (0.29, SE = 0.12, *p* = 0.001) groups. No difference emerged between the CA and MA groups. In summary, the WS group missed the target items the most compared to the MA and CA groups, which in turn missed more target items than the 5th graders and CS.

In the analyses of the incorrect type purchased phenomenon (i.e., only the category of drink) in the WS group and TD controls, the results of univariate analyses of variance revealed significant differences among groups [*F*(4,95) = 5.30, *p* = 0.001, η_p_^2^ = 0.18]. The WS and MA groups differed from the CS, 5th graders, and CA groups [WS, 1.15, SE = 0.19; MA, 0.85, SE = 0.19; CA, 0.15, SE = 0.19; 5th graders, 0.30, SE = 0.19; CS, 0.20, SE = 0.19]. No difference emerged between the MA and WS groups; no difference emerged among the CS, 5th graders, and CA groups.

To determine whether the 14 indices originated from a verbal or nonverbal nature, correlations of the 14 indices and the scores of block design were analyzed group-wise. The results revealed distinct patterns in each group. In the CS group, the payment duration and the total duration of pauses correlated with the block design scores; in the 5th grade group, none of the indices correlated with the block design scores. In the CA group, seven indices correlated with the scores of block design: the total length of distance, the total length of shopping distance from the first item shopped to a cashier counter, the total shopping duration, the total shopping duration from the first item shopped to a cashier counter, the duration of the first item shopped upon entrance, pauses during shopping, and the total duration of pauses. In the MA group, the total shopping duration, the total shopping duration from the first item shopped to a cashier counter, correct number of types purchased, and correct number of items purchased correlated with the block design scores. In the WS group, the total length of shopping distance from the first item shopped to a cashier counter, the duration of the first item shopped upon entrance, the correct number of types purchased, and the number of items purchased correlated with the block design scores. Almost all the indices were related to executive function, memory, and learning. Only purchased items and types were correctly or incorrectly related to language and semantic memory. Based on the correlation results, it was not easy to identify the nature of navigation verbally or non-verbally. In other words, the VR navigation task was related both verbally and non-verbally. Future studies could design pure verbal-based tasks compared to pure non-verbal-based tasks for people with neurodevelopmental disabilities. This study contributes to the evaluation of the semantic memory of people with WS and potential rehabilitation in cognition and language in the future.

## 6 Semantic organization of people with Williams syndrome

Based on the findings regarding the errors made by people with WS, it was hypothesized that people with WS processed items with bizarre semantic features. That is, the semantic organization (i.e., relatedness of lexical semantics or semantic groupings of relatedness in memory) of people with WS is deficient or atypical compared to TD controls. To identify the semantic features that may confound the identification of the target items for participants in navigation, 20 college students (10F/10M, mean age = 20.5, SD = 1.5, range = 18.11–24.7, see Table 1) were recruited to take part in a judgement task. Participants were required to name the images of the target items and misrecognized items on a questionnaire. After naming the items, participants wrote down the semantic features shared by the target items and misrecognized items in their minds. A target item could be paired with more than one misrecognized item, as long as participants mistakenly perceived them. On the questionnaire sheet, participants answered the questions in pairs until all pairs that confounded them had responded. One hundred and seventy four pairs were included in the questionnaire. These pairs were actual errors collected from the TD groups of CA, MA, CS, 5th graders, and people with WS. After analyzing the written content from the 20 college students, 22 confounding features that might result in incorrect replacement and confusion were identified. Two-way analyses of variance with repeated measures of replacement and confusion were then conducted with semantic features as a within-participants factor and groups as a between-participants factor. The results revealed three major groupings and one mixed grouping based on the different patterns across groups.

In the analyses regarding to replacement errors, the results revealed an interaction between semantic features and groups [*F*(84,1995) = 9.15, *p* < 0.001, η_p_^2^ = 0.27]. The simple main effect was from group differences for each type (21 significant semantic features; one non-significant semantic feature [interior constituents]). Parallel to factor analysis aiming at extracting common features in perceiving the items across groups, three major groupings of the semantic features were identified on the basis of the patterns found across groups as Table 3 shows.

**Table 3:** Semantic features attributed to the errors of replacement of the five groups.

Groupings	Semantic features	Strategy of confusion				
		College	5th Grade	CA	MA	WS
External attributes	Shapes	16	38	26	38	69
	Sizes	10	28	22	28	46
	Colors	12	32	18	40	65
	Smells	1	5	3	9	18
Internal attributes	Textures	5	15	11	13	28
	Ingredients	8	11	11	17	33
	Compositions	5	9	7	9	31
	Tastes	5	14	9	14	35
	Places of production	2	8	6	6	16
Structural organization	Places of use	1	6	6	17	14
	Materials	1	7	7	20	14
Practical features	Packaging materials	5	18	10	25	25
	Functions	11	28	16	42	56
Others	Wrappings	7	15	10	17	25
	Interior colors	0	8	4	1	4

The first grouping was shapes, sizes, wrappings, forms, colors, ingredients, compositions, textures, and tastes. In this grouping, taking shapes as an example, the WS group erred most (7.65, SD = 5.887) among the groups [MA, 4.35, SD = 3.602; CA, 1.95, SD = 2.523; 5th graders, 1.70, SD = 1.342; CS, 0.80, SD = 0.616]. The MA group erred significantly more than other groups (all *p* < 0.001) and less than the WS group (*p* = 0.002).

The second grouping included functions, places of production, smells, packaging materials, and growing patterns. In this grouping, taking functions as an example, the WS group (5, SD = 3.509, all at *p* < 0.001) erred as the MA group (4, SD = 3.372, vs. CA, *p* < 0.001; vs. 5th graders, *p* = 0.001; vs. CS, *p* = 0.001), which were in turn higher than other TD groups (CA, 1.40, SD = 1.903; 5th graders, 1.30, SD = 0.979; CS, 0.75, SD = 0.550).

The third grouping included production methods, prices, ways of eating, and places of use. In this grouping, taking production methods as an example, the WS group erred more (0.95, SD = 1.356) than all other groups [MA, 0.15, SD = 0.366, *p* < 0.001; CA, 0.20, SD = 0.523, *p* = 0.005; 5th graders, 0.30, SD = 0.571, *p* = 0.001; CS, 0, *p* = 0.001]. No difference was observed among the other groups.

A grouping of others was defined due to the other three semantic features are distinct in their characteristics. In the semantic feature of materials, the WS group (2.10, SD = 1.971) erred most among all groups (MA, 1.2, SD = 1.576, *p* = 0.030; CA, 0.60, SD = 0.995, *p* < 0.001; 5th graders, 0.65, SD = 0.933, *p* = 0.001; CS, 0.15, SD = 0.366, *p* < 0.001). The MA group erred more than the CS group (*p* = 0.012). No difference emerged among other groups. Regarding the semantic features of tastes, the WS group (0.55, SD = 0.826) differed from the CS (0, *p* < 0.001), 5th graders (0.10, SD = 0.308, *p* = 0.007), and CA group (0.20, SD = 0.523, *p* = 0.036). Regarding the semantic features of interior colors, the WS group (0.65, SD = 0.813) differed from the CS group (0.15, SD = 0.366, *p* = 0.020), but no difference emerged from other groups (5th graders, 0.30, SD = 0.470; CA, 0.25, SD = 0.444; MA, 0.80, SD = 1.005). The MA group erred similarly to the WS group but differed from the CS (*p* = 0.003), 5th graders (*p* = 0.020), and CA groups (*p* = 0.011).

To sum up, three major groupings of semantic features emerged, while replacements were considered. The first grouping is related to an external feature (i.e., shapes, sizes, wrappings, forms, colors, ingredients, compositions, textures, and tastes). The second grouping is related to the attached attributes (i.e., functions, places of production, smells, packaging materials, and growing patterns). The third grouping is related to a cuisine (i.e., production methods, prices, ways of eating, and places of use).

The main effect of group was significant [*F*(4,95) = 14.89, *p* < 0.001, η_p_^2^ = 0.38]. The WS group erred most (2.59, SE = 0.246) among groups (MA, 1.67, SE = 0.246, *p* = 0.010; CA, 0.70, SE = 0.246, *p* < 0.001; 5th graders, 0.61, SE = 0.246, *p* < 0.001; CS, 0.26, SE = 0.246, *p* < 0.001). The MA group erred more than other TD groups (CS, *p* < 0.001; 5th graders, *p* = 0.003; CA, *p* = 0.007) but less than the WS group (*p* = 0.010). The error patterns of the CS, 5th graders, and CA groups were similar in processing semantic features in replacement. The findings suggest that mature individuals replaced fewer targets and people with WS replaced more targets than most TD groups. However, the WS group appeared to be at the developmental stage, similar to the MA group. External features, attached attributes, and cuisines are influential semantic features for replacement. The WS group showed impaired processing of these semantic features, seeming to result in the purchase of the least correct target items. The frequency of errors in each semantic feature based on groupings across groups is presented in Table 3.

In the analyses regarding to confusion errors, the results revealed the interaction of types and groups [*F*(84,1995) = 4.66, *p* < 0.001, η_p_^2^ = 0.16]. The simple main effect was group differences for each type. Fifteen semantic features that confused the participants reached significance, but the other seven semantic features were not significant across groups (i.e., interior constituents, forms, growth patterns, production methods, prices, tastes, and ways of eating). Among the fifteen semantic features, four groupings emerged based on the difference patterns.

The first grouping included shapes, sizes, colors, and smells. In this grouping, take shapes as an example, the WS group confused most (3.45, SD = 2.114) than other groups (MA, 1.90, SD = 1.619, *p* = 0.002; CA, 1.30, SD = 1.129, *p* < 0.001; 5th graders, 1.90, SD = 1.518, *p* = 0.002; CS, 0.80, SD = 1.056, *p* < 0.001). The 5th graders and MA group (*p* = 0.026) were more confused than the CS (*p* = 0.026).

The second grouping included textures, ingredients, compositions, tastes, and places of production. In this grouping, take textures as an example, the WS group confused most (1.40, SD = 1.188) among groups (MA, 0.65, SD = 0.933, *p* = 0.008; CA, 0.55, SD = 0.686, *p* = 0.003; 5th graders, 0.75, SD = 0.851, *p* = 0.020; CS, 0.25, SD = 0.550, *p* < 0.001). No difference emerged among other groups.

The third grouping included the places of use and materials. For example, in this grouping, the WS group (0.70, SD = 0.979) confused more than the CS (0.05, SD = 0.224, *p* = 0.007). The MA group (1, SD = 1.124) confused more than the CS (0.05, SD = 0.224, *p* < 0.001), 5th graders (0.35, SD = 0.489, *p* = 0.007), and CA group (0.35, SD = 0.489, *p* = 0.007).

The fourth grouping consisted of packaging materials and functions. For example, in this grouping, take functions as an example, the WS group (2.80, SD = 2.285) confused more than the CS (0.55, SD = 0.686, *p* < 0.001), 5th graders (1.40, SD = 1.095, *p* = 0.004), and CA group (0.80, SD = 0.834, *p* < 0.001). The MA group (2.10, SD = 1.917) was more confused than the CS (*p* = 0.002) and CA (*p* = 0.007) groups.

The semantic features of the wrappings and interior colors were distinct in their characteristics and were listed as other grouping. In the semantic feature of wrappings, the WS group (1.25, SD = 0.910) confused more than the CS (0.35, SD = 0.489, *p* = 0.001) and CA groups (0.50, SD = 0.607, *p* = 0.005). No difference emerged between the 5th graders and MA group. In the semantic feature of interior colors, the WS group (0.20, SD = 0.080) and the CA group (0.20, SD = 0.080) yielded no difference from other groups. The CS (0, SD = 0.080, *p* = 0.001) and MA groups (0.05, SD = 0.080, *p* = 0.003) were less confused than the 5th graders (0.40, SD = 0.080).

In summary, four groupings of semantic features emerged, while confusion occurred. The first grouping is related to external attributes (i.e., shapes, sizes, colors, and smells). The second grouping is related to internal attributes (i.e., textures, ingredients, compositions, and places of production). The third grouping is related to structural organization (i.e., places of use and materials). The forth grouping is related to practical attributes (i.e., packaging materials and functions). Wrappings and interior colors were grouped as other factors which could confuse participants in purchasing the target items.

The main effect of group reached significance [*F*(4,95) = 9.16, *p* < 0.001, η_p_^2^ = 0.27], suggesting the WS group (1.20, SE = 0.119) confused most among groups (MA, 0.736, SE = 0.119, *p* = 0.007; CA, 0.450, SE = 0.119, *p* < 0.001; 5th graders, 0.623, SE = 0.119, *p* = 0.001; CS, 0.239, SE = 0.119, *p* < 0.001). The 5th graders and MA group (p = 0.004) were in turn more confused than the CS group (*p* = 0.025). Put together, the error patterns in processing semantic features in confusion revealed that the WS group confused most and the CS group confused least. That is, external attributes, internal attributes, structural organization, and practical attributes are influential semantic features that cause confusion. The WS group showed impaired processing of these semantic features, hence leading to the purchase of the most extra items under correct types. The frequency of errors in each type based on grouping across groups is presented in Table 4.

**Table 4:** Semantic features attributed to the errors of confusion of the five groups.

Groupings	Semantic Features	Groups				
		College	5^t^h Grade	CA	MA	WS
External features	Shapes	16	34	39	87	153
	Sizes	16	27	34	77	118
	Wrappings	13	26	29	62	93
	Forms	4	10	13	37	58
	Colors	8	28	31	79	122
	Ingredients	8	14	21	43	70
	Compositions	1	3	9	19	42
	Textures	3	15	13	31	50
	Tastes	5	20	20	47	70
Attached attributes	Functions	15	26	28	80	100
	Places of production	2	3	3	15	12
	Smells	1	3	2	26	29
	Packaging materials	13	20	23	54	75
	Growing patterns	0	1	1	10	7
Cuisines	Production methods	0	6	4	3	19
	Prices	0	0	0	1	6
	Ways of eating	0	5	7	7	21
	Places of use	0	1	2	5	15
Others	Materials	3	13	12	24	42
	Interior colors	3	6	5	16	13
	Flavors	0	2	4	5	11

## 7 Validity of the virtual navigation task

To make sure whether a practice effect emerged in navigation, four shopping malls with three purchasing tasks were tested. Two-way analysis of variance with the within-participants factor and the group as the between-participants factor was conducted. The results revealed that the CA and MA controls showed clear practice effects by navigating the virtual supermarket from 1st to 4th and plateaued at the 3rd (i.e., CA, MA patterns); the WS group showed a practice effect at a slower pace to the 4th and kept learning through navigations. The practice effect on the 4th index across the four navigation tasks in the five groups is listed in Table 5.

**Table 5:** The practice effect shown in navigation.

No.	Index	Mall × Group interaction^a^	Group main effect^b^	Mall main effect^c^
1	The total length of distance in meters from entrance to exit	1.612, *p* = 0.146	6.982, *p* = 0.002	15.385, *p* < 0.001
2	The total length of shopping distance in meters from the first item selected to a cashier counter	1.76, *p* = 0.11	5.596, *p* = 0.006	13.625, *p* < 0.001
3	The total shopping duration in seconds from entrance to exit	2.412, *p* = 0.029	21.403, *p* < 0.001	69.574, *p* < 0.001
4	The total shopping duration from the first item selected to a cashier counter	2.397, *p* = 0.03	20.03, *p* < 0.001	66.617, *p* < 0.001
5	The payment duration from the cost displayed on the screen to the purchasing icon clicked	1.747, *p =* 0.113	0.097, *p* = 0.908	0.783, *p* = 0.505
6	The duration of the first item shopped upon entrance	1.543, *p =* 0.167	18.627, *p* < 0.001	10.131, *p* < 0.001
7	Correct number of types purchased	0.296, *p =* 0.938	11.664 *p* < 0.001	0.875, *p* = 0.455
8	Correct number of items purchased	0.536, *p =* 0.78	9.986, *p* < 0.001	1.305, *p* = 0.274
9	Incorrect number of types purchased	0.891, *p =* 0.503	17.765, *p* < 0.001	0.692, *p* = 0.558
10	Incorrect number of items purchased	0.947, *p =* 0.463	18.782, *p* < 0.001	0.651, *p* = 0.584
11	Pauses during shopping	0.999, *p* = 0.428	10.506, *p* < 0.001	73.21, *p <* 0.001
12	The total duration of pauses	2.877, *p* = 0.011	14.151, *p* < 0.001	55.015, *p <* 0.001
13	Number of incorrect actions	0.843, *p* = 0.538	0.769, *p* = 0.468	2.484, *p =* 0.062
14	Warning reminders of errors	0.686, *p* = 0.661	1.122, *p* = 0.333	2.572, *p =* 0.056

^a^*F*_(6, 171)_ for mall × group interaction, ^b^*F*_(2, 57)_ for group main effect, ^c^*F*_(3, 171)_ for mall main effect.

An interaction of malls and groups reached significance in three indices (the total shopping duration in seconds from entrance to exit, *F*(6,171) = 2.412, *p* = 0.029; the total shopping duration from the first item shopped to a cashier counter, *F*(6,171) = 2.397, *p* = 0.03; the total duration of pauses, *F*(6,171) = 2.877, *p* = 0.011. Each index across groups was analyzed accordingly.

In the index of total shopping navigation duration from entrance to exit, the simple main effect was the mall effect in each group. In the CA group, the total duration was significantly different from 1st to 4th navigations supermarket-wised (1st, 422, SD = 162; 2nd, 320, SD = 102; 3rd, 288, SD = 81; 4th, 271, SD = 79; 1 vs. 2, *p* < 0.001; 1 vs. 3, *p* < 0.001; 1 vs. 4, *p* < 0.001; 2 vs. 3, *p* = 0.031, 2 vs. 4, *p* = 0.007; 3 vs. 4, ns). The CA group reached their plateau in navigation from 3rd to 4th. In the MA group, the total navigation duration was significantly different from 1st to 4th navigations (1st, 529, SD = 174; 2nd, 438, SD = 139; 3rd, 370, SD = 90; 4th, 342, SD = 123; 1 vs. 2, *p* = 0.001, 1 vs. 3, *p* < 0.001, 1 vs. 4, *p* < 0.001; 2 vs. 3, *p* = 0.006, 2 vs. 4, *p* < 0.001; 3 vs. 4, ns). The MA group also reached their plateau in navigation from 3rd to 4th. In the WS group, the total navigation duration continued learning from 1st to 4th (1st, 683, SD = 156; 2nd, 581, SD = 147; 3rd, 522, SD = 157; 4th, 420, SD = 83; 1 vs. 2, *p* = 0.001, 1 vs. 3, *p* < 0.001, 1 vs. 4, *p* < 0.001; 2 vs. 3, ns, 2 vs. 4, *p* < 0.001; 3 vs. 4, *p* = 0.005).

Another simple main effect was the group effect for navigations in each mall [1st, *F*(2,57) = 12.60, *p* < 0.001, η_p_^2^ = 0.30; 2nd, *F*(2,57) = 19.87, *p* < 0.001, η_p_^2^ = 0.41; 3rd, *F*(2,57) = 21.32, *p* < 0.001, η_p_^2^ = 0.42; 4th, *F*(2,57) = 11.52, *p* < 0.001, η_p_^2^ = 0.28]. In each virtual navigation, the CA group showed a significantly shorter processing duration than the MA and WS groups, while the MA group showed a significantly shorter processing duration than the WS group.

In the index of the total shopping duration from the first item shopped to a cashier counter, the simple main effect was the navigation effect of malls in each group. In the CA group [*F*(3,57) = 19.20, *p* < 0.001, η_p_^2^ = 0.50], the total shopping duration from the first item shopped to a cashier counter was significantly different from 1st to 4th navigations (1st, 385, SD = 149; 2nd, 287, SD = 90; 3rd, 252, SD = 76; 4th, 247, SD = 75; 1 vs. 2, *p* < 0.001, 1 vs. 3, *p* < 0.001, 1 vs. 4, *p* < 0.001; 2 vs. 3, *p* = 0.012, 2 vs. 4, *p* = 0.020; 3 vs. 4, ns). In the MA group [*F*(3,57) = 25.24, *p* < 0.001, η_p_^2^ = 0.57], the total shopping duration from the first item shopped to a cashier counter was significantly different from 1st to 4th navigations (1st, 476, SD = 154; 2nd, 393, SD = 126; 3rd, 333, SD = 77; 4th, 305, SD = 103; 1 vs. 2, *p* = 0.001, 1 vs. 3, *p* < 0.001, 1 vs. 4, *p* < 0.001; 2 vs. 3, *p* = 0.010, 2 vs. 4, *p* < 0.001; 3 vs. 4, ns). In the WS group [*F*(3,57) = 25.50, *p* < 0.001, η_p_^2^ = 0.57], the total shopping duration from the first item shopped to a cashier counter was significantly different from 1st to 4th navigations (1st, 608, SD = 142; 2nd, 512, SD = 135; 3rd, 470, SD = 151; 4th, 370, SD = 80; 1 vs. 2, *p* = 0.001, 1 vs. 3, *p* < 0.001, 1 vs. 4, *p* < 0.001; 2 vs. 3, ns; 2 vs. 4, *p* < 0.001; 3 vs. 4, *p* = 0.004).

Another simple main effect was the group effect on each mall with the times of navigation [1st, *F*(2,57) = 11.37, *p* < 0.001, η_p_^2^ = 0.28; 2nd, *F*(2,57) = 17.80, *p* < 0.001, η_p_^2^ = 0.38; 3rd, *F*(2,57) = 20.83, *p* < 0.001, η_p_^2^ = 0.42; 4th, *F*(2,57) = 9.96, *p* < 0.001, η_p_^2^ = 0.25]. In each navigation, the CA group showed a significantly shorter processing duration than the MA and WS groups; the MA group showed a significantly shorter processing duration than the WS group. In the index of the total duration of pauses in seconds, the simple main effect was the navigations in malls in each group. In the CA group [*F*(3,57) = 15.78, *p* < 0.001, η_p_^2^ = 0.45], the total duration of pauses was significantly different from 1st to 4th navigations supermarket-wised (1st, 385, SD = 149; 2nd, 287, SD = 90; 3rd, 252, SD = 76; 4th, 247, SD = 75; 1 vs. 2, *p* = 0.001, 1 vs. 3, *p* = 0.001, 1 vs. 4, *p* < 0.001; 2 vs. 3, *p* = 0.022, 2 vs. 4, *p* = 0.001; 3 vs. 4, *p* = 0.031). In the MA group [*F*(3,57) = 27.04, *p* < 0.001, η_p_^2^ = 0.58], the total duration of pauses was significantly different from 1st to 4th navigations (1st, 200, SD = 113; 2nd, 157, SD = 100; 3rd, 119, SD = 65; 4th, 104, SD = 76; 1 vs. 2, *p* < 0.001, 1 vs. 3, *p* < 0.001, 1 vs. 4, *p* < 0.001; 2 vs. 3, *p* = 0.008, 2 vs. 4, *p* < 0.001; 3 vs. 4, ns). In the WS group [*F*(3,57) = 18.46, *p* < 0.001, η_p_^2^ = 0.49], the total duration of pauses was significantly different from 1st to 4th navigations (1st, 288, SD = 143; 2nd, 222, SD = 113; 3rd, 197, SD = 109; 4th, 158, SD = 72; 1 vs. 2, *p* = 0.001, 1 vs. 3, *p* = 0.001, 1 vs. 4, *p* < 0.001; 2 vs. 3, ns; 2 vs. 4, *p* < 0.001; 3 vs. 4, *p* = 0.008).

Another simple main effect was from the group effect in navigations of each mall [1st, *F*(2,57) = 11.42, *p* < 0.001, η_p_^2^ = 0.28; 2nd, *F*(2,57) = 12.33, *p* < 0.001, η_p_^2^ = 0.30; 3rd, *F*(2,57) = 14.12, *p* < 0.001, η_p_^2^ = 0.33; 4th, *F*(2,57) = 12.04, p < 0.001, η_p_^2^ = 0.29]. In each navigation, the CA group showed a significantly shorter total duration of pauses in seconds compared to the MA and WS groups; the MA group showed a significantly shorter total duration of pauses in seconds than the WS group.

## 8 Discussion

To evaluate the nature of semantic memory of people with WS and examine the potential improvement in semantic memory through navigations in people with WS, a virtual navigation task with supermarket setting was conducted. Fourteen indices originating from studies on people with MCI related to memory, learning, language, executive function, and attention were measured. To obtain typically developing patterns in virtual navigation, CS and 5th graders were recruited. Analyses of the control groups revealed that the CS and 5th graders were faster and erred less in completing the task than the CA controls did. Analyses of the WS and TD groups showed that the WS group was the slowest and erred more in completing the task than the CA and MA groups did. These findings suggest that the navigation task is a valid tool for differentiating developmental stages with varying degrees of intellectual abilities. Further analyses of the error patterns revealed group differences in the extra items purchased under the correct types and in the correct items missed. The 5th graders erred more than the CS on the extra items purchased under correct types; the CA group erred more than the CS and 5th graders on the correct items missed. The WS group erred more than the CA and MA groups on incorrect items purchased under the correct types, extra items purchased under the correct types, correct items missed, and incorrect types purchased. The findings revealed that the MA group showed CA-like error patterns on incorrect items purchased under correct types, extra items purchased under correct types, and correct items missed, but differed from the CA group on incorrect types purchased.

Further analyses of strategies and phenomena while making errors in virtual navigation revealed that the WS group confused the categories of fruits and vegetables more than the CA and MA groups did. Moreover, the WS group made more replacement, extra-buying, and correct items missed than the CA and MA groups in the overall categories.

Semantic features attributed to replacement and confusion were further analyzed to determine the potential atypical processing of people with WS. In the error patterns of replacement, the semantic features of the external features, attached attributes, and cuisines were influential factors. In the error patterns of confusion, the semantic features of the external attributes, internal attributes, structural organization, and practical attributes were risk factors. Owing to the atypical processing of these semantic features, the WS group replaced the target items from various distractors and was confused with other items displayed in the virtual navigation tasks. Hence, the WS group erred the most and purchased extra items even under the correct types. Consistent with a previous study on false memory tasks in people with Down syndrome (Hsu et al., 2024), the current study revealed that the semantic organization of people with WS is atypical, resulting in bizarre lexical semantics in their memory. According to the parallel distributed processing model (McClelland and Cleeremans, 2009; Rumelhart et al., 1986), the input layer of people with WS is wary, leading to atypical outputs in language processing and cognitive functions. The current study further contributes to determining possible semantic features that may be confused and replaced in people with WS. Owing to the atypical semantic features of lexical items, semantic groupings might be deficient, as the spreading activation model reveals (Collins and Loftus, 1975).

The practice effect was confirmed across groups on the total duration of navigation from entrance to exit, the total duration of navigation from the first item purchased, and the total duration of pauses, though people with WS were still in the learning process. This effect indicated potential rehabilitation using VR in people with WS, as the hypothesis of brain and behavior asymmetry reveals, although the effect seemed to be shown in executive function, learning, and attention rather than in language and memory per se. These results are consistent with the deficient long-term memory of semantic knowledge observed in people with WS.

In the current study, all analyses aimed to examine the semantic memory of people with WS and to determine the root cause of bizarre semantic processing in contextual integration and sentence comprehension. In the navigation tasks, people with WS and the TD controls were required to complete the shopping tasks from 1st to 4th navigations. These navigation tasks are related to the evaluation of semantic memory rather than visuospatial perception per se. This navigation task was both verbal and visuospatial related. Hence, it was impossible to recruit a control group that matched the visuospatial ability of people with WS only. The best way to recruit control groups was to match CA and MA to people with WS, which was believed to be the most appropriate and best design for this study. The MA group was generated using intellectual tests. In the future, a nonverbal control navigation task without semantic involvement could be conducted and compared to the current study to determine the correlation of navigation tasks with verbal and nonverbal nature. The correlation could also reveal whether the practice effect of navigation results from verbal or nonverbal causes.

## 9 Conclusion

Using VR, the nature of semantic memory abilities in people with WS was identified. VR is a useful tool for assessing the memory of people with neurodevelopmental disabilities (Ge et al., 2018), as the current study revealed in people with WS. Moreover, the practice effect with times in navigating virtual supermarkets was confirmed in people with WS. Although the effect was mainly on the total duration of navigation and pauses during the execution of shopping tasks, the potential for rehabilitation of people with WS is promising. People with WS were still learning through navigation. More effort should be devoted to studies using VR as an intervention tool for people with WS.

In the analyses of the error patterns of people with WS and the TD controls, significant errors in replacement and confusion made by people with WS were observed compared to the CA and MA groups. Further analyses revealed the processing of bizarre semantic features in each group. Bizarre semantic features are defined as atypical inputs related to the concept of a lexical item. The atypical processing of semantic features in people with WS might be the root cause of deficient semantic organization. The educational implications rely on teaching lexical items in their richness and depth by utilizing contextual cues to people with WS.

VR technology is compatible with constructivist learning theory, which integrates components of cognitive abilities such as attention, memory, executive function, and language (Chen, 2009). VR technology can be developed by defining problems, designing platforms, developing hardware with software, and evaluating technology. With this effort, people with WS can enhance their abilities in daily life and become more inclusive of their surroundings. More daily surroundings with high familiarity can be designed, such as gymnasiums, fast-food restaurants, and aquariums, and tested on people with WS. A more advanced design of VR settings could be used and tested to make the contents and environments more dynamic and immersive for participants. Using VR technology, which is very close to daily life, people with neurodevelopmental disabilities can benefit from this technology. More influential factors should be considered in future studies using VR techniques, such as goggles, to make navigation immersive and handles or mouse to control the involvement of attention and execution functions. The current study was conducted in a non-immersive VR setting. Further studies could incorporate real-life supermarket navigation to compare the results of VR studies across groups to make the VR technique a truly promising tool for people with neurodevelopmental disabilities in the future.

## Data Availability

The original contributions presented in the study are included in the article/supplementary material, further inquiries can be directed to the corresponding author.
